# Sodium nitroprusside is not associated with metabolic acidosis during intraoperative infusion in children

**DOI:** 10.1186/1471-2253-13-9

**Published:** 2013-04-30

**Authors:** Gregory B Hammer, Sara G Connolly, Scott R Schulman, Andrew Lewandowski, Carol Cohane, Tammy L Reece, Ravinder Anand, Jeff Mitchell, David R Drover

**Affiliations:** 1 Department of Anesthesia, Stanford University School of Medicine, Stanford, USA; 2 Department of Anesthesiology, Stanford University School of Medicine, Stanford, USA; 3 Duke University School of Medicine, Rockville, MD, USA; 4 Duke Clinical Research Institute, Rockville, MD, USA; 5 Emmes Corporation, Rockville, MD, USA

## Abstract

**Background:**

Sodium nitroprusside (SNP) is a potent vasodilator that has been used to induce deliberate hypotension in children during surgery involving significant blood loss, including craniofacial and spinal fusion procedures. SNP metabolism liberates cyanide, which may cause interference with cellular energy metabolism, leading to metabolic acidosis and central nervous system injury. We performed a retrospective, case–control study to determine whether the short-term intra-operative use of SNP for deliberate hypotension is associated with metabolic acidosis in children undergoing surgical procedures for craniofacial or spinal anomalies. Cyanide and thiocyanate concentrations were also recorded in patients who received SNP.

**Methods:**

Data from 166 children undergoing craniofacial and spinal fusion surgery between 2005 and 2010 at Lucile Packard Children's Hospital (LPCH) at Stanford were analyzed. Records from 60 patients who received SNP (SNP group) as part of a multicenter, randomized, double-blind study were compared with records from 106 eligible patients who had blood pressure reduction using anesthetic agents and did not receive SNP (control group). Metabolic acidosis was defined as serum bicarbonate (HCO_3_) < 18.5 mEq/L. Whole blood CN, plasma thiocyanate and urinary thiocyanate concentrations were measured in patients in the SNP group. Differences in metabolic acidosis rates between the SNP and control groups were assessed through a test of noninferiority in the rate for the SNP group with a noninferiority threshold of 0.2. A z-test was used to test the null hypothesis. The alternative hypothesis was that the difference in these rates was less than 0.2. The same noninferiority threshold of 0.2 was also used to perform separate, secondary tests for noninferiority in the proportion of patients with HCO_3_ levels below 18.5 mEq/L and the proportion of patients who required HCO_3_ administration.

**Results:**

Fewer patients in the SNP group experienced metabolic acidosis compared to the control group (31.7% vs. 36.8%, respectively; p < .001). No whole blood CN levels above the lower limit of quantification were detected in any of the 51 patients with validated CN data. Plasma and urinary thiocyanate levels were also low.

**Conclusions:**

Our findings suggest that SNP, when used for short-term deliberate hypotension, does not cause an increased incidence of metabolic acidosis compared with the use of anesthetic agents alone.

**Trial registration:**

Trial registration number: NCT00135668

## Background

**Figure 1:**
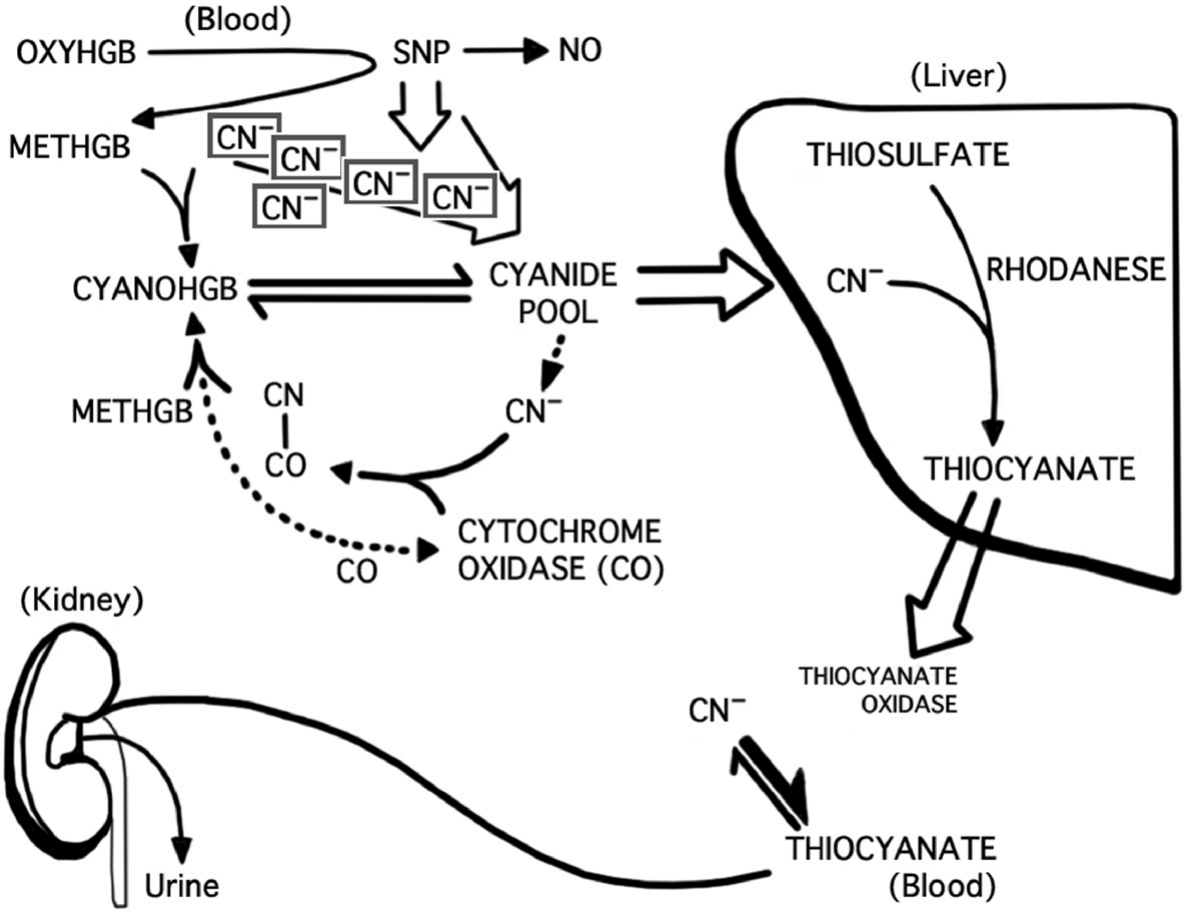
SNP metabolism. ^*^SNP = sodium nitroprusside; CN^-^ = cyanide; NO = nitric oxide; METHGB = methemoglobin; CYANOHGB = cyanohemoglobin; OXYHGB = oxyhemoglobin. ^*^Modified with permission from Figure 1[2].

Sodium nitroprusside (SNP) is a potent vasodilator that has been used to induce deliberate hypotension in children during surgery involving significant blood loss, including craniofacial and spinal fusion procedures. The benefits of SNP include rapid onset and offset, i.e. titratability. A potential drawback of SNP is that its metabolism results in the liberation of cyanide ions (CN). Cyanide, in turn, may cause interference with cellular energy metabolism, leading to metabolic acidosis and central nervous system injury. Infants and children treated with SNP may be at increased risk for toxicity due to immature enzyme systems or lack of thiosulfate stores, as thiosulfate is important in the detoxification of CN via conversion to thiocyanate (Figure 1) [1]. Signs of cyanide toxicity, including delirium, weakness, vomiting and coma, may be masked by general anesthesia. Although data describing the relationship between SNP and metabolic acidosis during anesthesia are limited, concern of the development of metabolic acidosis due to cyanide toxicity may decrease the use of SNP during general anesthesia.

We performed a retrospective case–control study to determine whether the short-term intra-operative use of SNP for deliberate hypotension is associated with metabolic acidosis in children undergoing craniofacial or spinal surgery. Cyanide and thiocyanate concentrations were also recorded in patients who received SNP.

## Methods

After approval from the Institutional Review Board was obtained, data from 166 children undergoing craniofacial and spinal fusion surgery between 2005 and 2010 at Lucile Packard Children’s Hospital (LPCH) at Stanford were analyzed. Records from 60 patients at LPCH who received SNP (SNP group) as part of a multicenter, randomized, double-blind study were compared with historical records from 106 eligible patients who had blood pressure reduction using anesthetic agents and did not receive SNP (control group). The degree of blood pressure reduction for each patient was determined by their anesthesia care providers.

Patients in the SNP treatment group were treated as part of the protocol NICHD-2003-09-DR. All patients treated at LPCH who met the eligibility requirements were included in this analysis. Enrollment for this study began on August 3, 2005, and the last participant completed on January 10, 2008. Per protocol, patients were randomized to receive 30 minutes of blinded SNP infusion at 0.3, 1, 2, or 3 mcg/kg/min following initial stabilization of anesthesia. Open-label drug administration was initiated at the completion of the blinded infusion, during which time SNP was titrated to achieve a MAP lower than baseline determined by the investigator. Minimum values for MAP were 50 mmHg for patients > 1 month of age and 40 mmHg for neonates. The open-label phase was completed when the need for blood pressure control or general anesthesia was terminated.

Data for patients in the control group were collected retrospectively related to surgeries performed from May 18, 2005 to January 22, 2010. All data were procured from the LPCH electronic medical records system following completion of enrollment in the SNP group. Data included demographics, anesthesia records (including intravenous fluids, estimated blood loss and volume of blood administered as packed red blood cells) and laboratory results.

Patients eligible for this analysis had corrective surgery for diagnoses of craniosynostosis or scoliosis. Inclusion criteria also included weight ≥ 3 kg and age < 17 years at the time of surgery. Patients were excluded from the control group if they received SNP during or within 6 hours after the surgical procedure. Additional inclusion criteria for the NICHD-2003-09-DR protocol were that neonates needed to be full-term gestation, participants needed to require pharmacologically-induced hypotension for acute blood pressure management during surgery, the duration of controlled hypotension was expected to be at least 2 hours, the participant required general anesthesia with endotracheal intubation or IV sedation without endotracheal intubation, and informed consent was required from the parent or legal guardian and, when appropriate, the participant. Additional exclusion criteria for the NICHD-2003-09-DR protocol were a known allergy to SNP, mitochondrial cytopathy with a disorder of oxidative phosphorylation or respiratory chain enzymes, a contraindication to vasodilator therapy for blood pressure control, a positive screening urine or serum HCG result, participation in clinical trials within 30 days before enrollment, a serious medical condition which would interfere with study procedures, or likely death within 48 hours.

Metabolic acidosis was defined as serum bicarbonate (HCO_3_) < 18.5 mEq/L as measured by arterial blood gas tension analysis and/or administration of HCO_3_ during anesthesia. Patients in the SNP group had arterial blood gas tensions recorded hourly (± 10 minutes) after the initiation of SNP administration or when an adverse event was observed. Patients in the control group did not have regular arterial blood gas sampling; serum bicarbonate values for all samples measured in the control group were evaluated. Patients were removed from the analysis if no HCO_3_ measurements were recorded during anesthesia administration.

Historical records from 126 patients who satisfied the inclusion and exclusion criteria were screened for inclusion in the control group. This number corresponded to the time frame we chose (2005–10), bracketing enrollment of treatment group patients, that we anticipated would be needed in order to be left with a sufficient number of control patients once those in whom no bicarbonate was checked or administered were excluded. After eliminating patients in whom HCO_3_ was not measured during anesthesia, 106 of 126 screened patients were included in the control group for this analysis. Eighty participants were treated with SNP at LPCH under the NICHD-2003-09-DR protocol. Twenty of these 80 participants were excluded from the SNP treatment group for this analysis since they were treated for conditions unrelated to scoliosis or craniosynostosis.

Differences in metabolic acidosis rates between the SNP and control groups were assessed through a test of noninferiority in the rate for the SNP group. A noninferiority threshold of 0.2 was chosen before retrospective data for the control group were collected. A z-test was used to test the null hypothesis that the metabolic acidosis rate for the SNP group was at least 0.2 greater than the rate in the control group. The alternative hypothesis was that the difference in these rates was less than 0.2. The same noninferiority threshold of 0.2 was also used to perform separate, secondary tests for noninferiority in the proportion of patients with HCO_3_ levels below 18.5 mEq/L and the proportion of patients who required sodium bicarbonate administration.

In addition, two-sided tests were performed to assess differences in demographic, baseline, and operative characteristics between the SNP and control groups. Fisher’s exact tests were used for categorical variables, and the nonparametric Kruskal-Wallis test was used for continuous variables. No multiplicity adjustment was performed.

The study was originally powered to have 80% power with 125 participants in the control group and a true metabolic acidosis rate above 42%. This power calculation used the assumption that data from 77 patients would be included in the SNP group and that the metabolic acidosis rate is 44% in this group. Thus, the sample sizes used in the analysis are less than the sizes suggested by the power calculation. However, as seen in the results section, the observed metabolic acidosis rates in both groups were considerably lower than the assumed rates.

Data for the control group were collected beyond the January 8, 2008 end of enrollment in the SNP group in order to achieve 80% power. Thirty-eight (36%) of the 106 patients in the control group had surgery after enrollment in the control group closed.

Blood samples used to determine whole blood CN and plasma thiocyanate concentrations for the SNP group were scheduled to be drawn once before SNP infusion began, once after SNP administration was terminated, and up to four times during SNP administration. Urinary thiocyanate measurements were also performed from samples obtained every 4 hours after SNP was initiated. Maximum CN and thiocyanate concentrations are summarized. Nine of the 60 patients did not have usable cyanide or thiocyanate concentrations due to initial use of an assay that could not be validated.

### Patient characteristics and operative information

As shown in the demographic summary in Table 1, the SNP group consisted of 40 patients undergoing craniofacial surgery (66.7%) and 20 patients having spinal fusion surgery (33.3%). In the control group, 71 patients underwent craniofacial surgery (67.0%) and 35 (33.0%) had spinal fusion surgery. Average durations of anesthesia for the SNP and control groups were 327 and 309 minutes, respectively. Demographic characteristics were similar between the two treatment groups.

**Table 1:** Demographics

	**SNP (N=60)**	**Control (N=106)**	**P Value**
**Diagnosis**			
Craniosynostosis and Craniofacial Anomalies	40 (66.7%)	71 (67.0%)	1.0
Scoliosis and Spinal Anomalies	20 (33.3%)	35 (33.0%)	
**Age (Months)**			
Mean (SE)	79.4 (10.2)	61.9 (7.4)	0.30
Median (Min, Max)	26.0 (2.0, 203.0)	10.5 (3.0, 191.0)	
**Weight (kg)**			
Mean (SE)	24.4 (2.6)	23.7 (2.3)	0.76
Median (Min, Max)	13.7 (5.3, 73.0)	9.6 (5.3, 100.0)	
**Sex**			
Male	22 (36.7%)	49 (46.2%)	0.26
Female	38 (63.3%)	57 (53.8%)	
**Race**			
Asian	5 (8.3%)	12 (11.3%)	0.94
Black or African American	1 (1.7%)	3 (2.8%)	
White or Caucasian	51 (85.0%)	85 (80.2%)	
Multiracial or Other	3 (5.0%)	5 (4.7%)	
Not Reported	0	1 (0.9%)	

A summary of anesthetic agents administered according to treatment group is shown in Table 2. Fifty percent of patients in the SNP group received isoflurane compared to 53.8% in the control group. The proportion of patients receiving various intravenous anesthetic agents showed considerable differences between groups. Because there was no standardization of anesthetic technique in the control group, these differences are due to variability in the preferences of the anesthesia providers caring for each patient.

**Table 2:** Summary of anesthetics administered according to treatment group

	**SNP (N= 60)**	**Control (N= 106)**	**P Value**
Sevoflurane Administered	13 (21.7%)	74 (69.8%)	<0.001^*^
Isoflurane Administered	30 (50.0%)	57 (53.8%)	0.75
Halothane Not Administered	60 (100%)	106 (100%)	
Remifentanil Administered	29 (48.3%)	76 (71.7%)	0.004^*^
Propofol Administered	41 (68.3%)	25 (23.6%)	<0.001^*^
Fentanyl Administered	37 (61.7%)	0	<0.001^*^
Ketamine Administered	35 (58.3%)	4 (3.8%)	<0.001^*^

^*^ The summary of anesthetic agents is provided for completeness. The reasons for discrepancies between groups are not known.

Table 3 summarizes the total amount of SNP administered over the course of the study in mcg/kg, the average SNP infusion rate in mcg/kg, and the duration of infusion for patients in the SNP group. The mean total administered dose was 124.7 mcg/kg with a large standard deviation (114.3). The lowest total dose was 0.9 mcg/kg in a patient that was on SNP for 9 minutes, although no other participant had a total dose below 12.7 mcg/kg. The mean duration of infusion was 139.5 minutes with a standard deviation of 65.4 minutes. The maximum duration of infusion was 339 minutes. The average infusion rate was determined by dividing the total administered dose by the infusion duration. The mean of these rates was 0.9 mcg/kg/min. The maximum average infusion rate was 2.9 mcg/kg/min. During open-label drug administration, three participants received SNP at infusion rates that exceeded 3 mcg/kg/min, which was the highest infusion rate used during the blinded phase. The highest infusion rate used during the open-label portion of the study was 4.5 mcg/kg/min. One patient was administered SNP at this rate for 23 minutes.

**Table 3:** SNP infusion data for the 60 patients in the SNP group

**Total administered SNP dose (mcg/kg)**	
Mean (SD)	124.7 (114.3)
Median (Min-Max)	94.4 (0.9, 587.9)
**Duration of SNP administration (min)**	
Mean (SD)	139.5 (65.4)
Median (Min-Max)	141.0 (9.0, 339.0)
**Average SNP infusion rate (mcg/kg/min)**	
Mean (SD)	0.9 (0.6)
Median (Min-Max)	0.7 (0.1, 2.9)

## Results

### Blood loss and IV fluid and blood administration

There was no statistically significant difference in blood loss or IV fluid volume administered between the two groups. The average volume of blood administered was significantly greater in the SNP group (17.8 ± 2.0 mL/kg) compared to the control group (11.4 ± 1.3 ml/kg) (p<0.01) (Table 4).

**Table 4:** Duration of anesthesia, estimated blood loss and IV fluid and blood administration

	**Treatment (N=60)**	**Control (N=106)**	**P Value**
**Duration of anesthesia (min) (SE)**	327 (15.2)	309 (9.6)	0.45
**Estimated blood loss (ml/kg) (SE)**	19.9 (2.8)	18.9 (1.6)	0.94
**Volume of IV fluid administered (ml/kg) (SE)**	53.3 (5.3)	45.8 (3.2)	0.33
**Volume of blood administered (ml/kg) (SE)**	17.8 (2.04)	11.4 (1.3)	<0.001

### Metabolic acidosis

As shown in Table 5, fewer patients in the SNP group experienced metabolic acidosis compared to the control group (31.7% vs. 36.8%, respectively; p < .001). The 95% confidence interval for the difference between these percentages is (−22%, 11%). Many of the cases of metabolic acidosis in the control group were identified through HCO_3_ administration. A higher percentage of participants in the control group received HCO_3_ during anesthesia (30.2% compared to 16.7% in the SNP group).

**Table 5:** Metabolic acidosis summary by treatment group

	**SNP (N= 60)**	**Control (N= 106)**	**P Value**
**Metabolic acidosis**	19 (31.7%)	39 (36.8%)	<0.001
**Recorded HCO**_**3**_**< 18.5 mEq/L**	16 (26.7%)	16 (15.1%)	0.42
**Administered HCO**_**3**_	10 (16.7%)	32 (30.2%)	<0.001

More patients in the SNP group than in the control group had recorded HCO_3_ levels below 18.5 mEq/L (26.7% compared to 15.1%). However, the test for noninferiority does not show that the proportion in the SNP group is significantly greater (p-value = 0.423). Also, participants in the SNP group tended to have more collected HCO_3_ measurements than participants in the control group. The median number of collected measurements was 5 (lower quartile = 3) and the median in the control group was 3 (lower quartile = 2).

Also, 16 (42%) of the 38 patients in the control group who had surgery after the SNP study had closed in January 2008 had MA. This percentage was slightly higher than the percentage (34%) of the 68 patients in the overall control group with MA and surgery before 2008.

**Figure 2:**
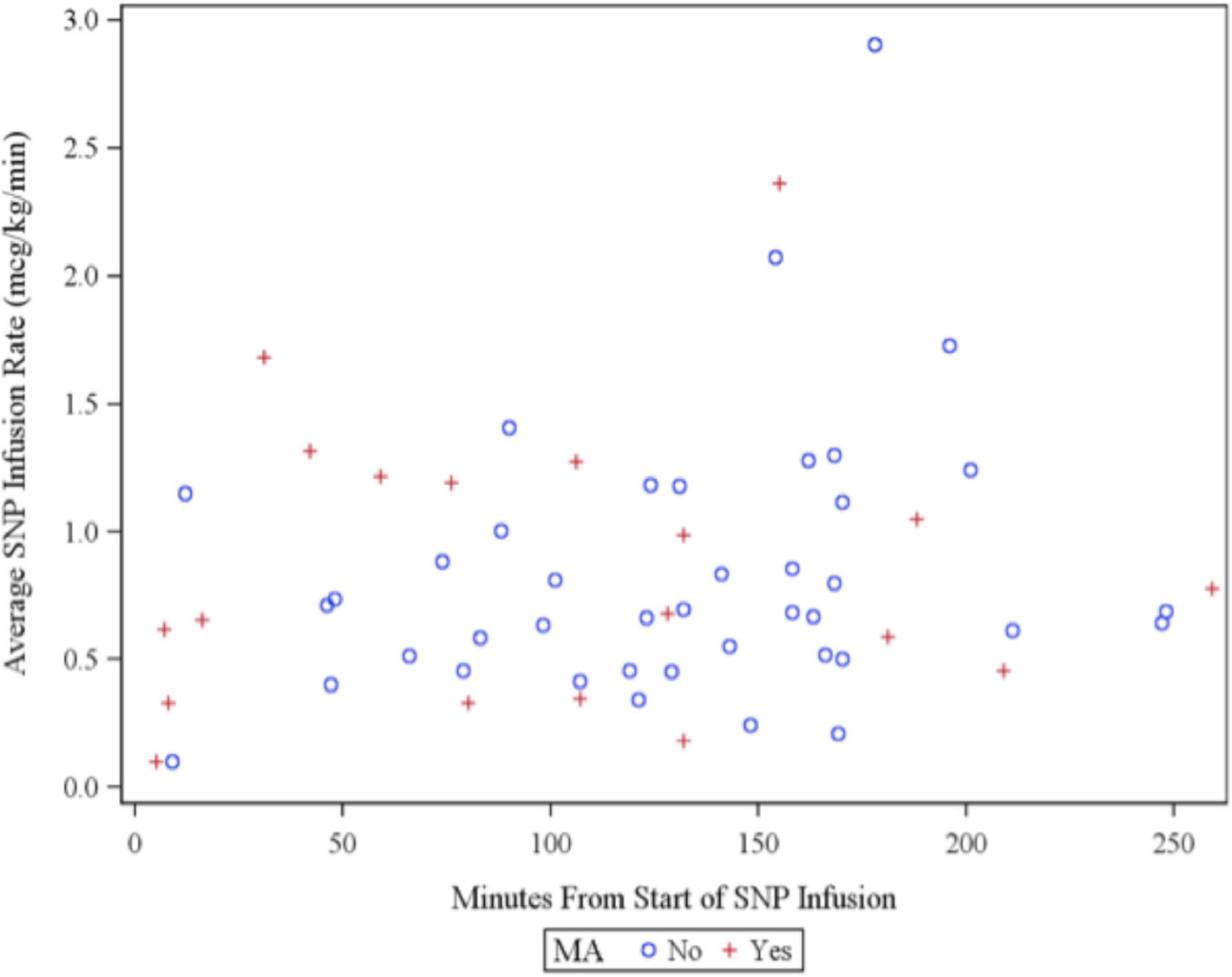
**Plot of relationship between metabolic acidosis (MA) occurrence and both the duration of SNP administration and average SNP infusion rate until either MA was detected or the end of the study.** Only patients in the SNP group are plotted. Patients with and without MA are plotted using a “+” and “o,” respectively.

In order to explore whether patients in the SNP group who received higher average SNP infusion rates or higher total amounts of SNP were more likely to have experienced metabolic acidosis, the duration of SNP administration and average infusion rate until either metabolic acidosis was first identified or the end of study were examined (Figure 2). Patients are identified in the plot as either having or not having metabolic acidosis. There is not a strong relationship between dose and metabolic acidosis. One (33.3%) of the 3 patients with an average infusion rate greater than 2 mcg/kg/min experienced metabolic acidosis. Seven (36.8%) of the 19 patients with an infusion rate greater than 1 mcg/kg/min experienced metabolic acidosis. Both percentages are similar to the metabolic acidosis rates in either the overall SNP group or the control group.

### Cyanide and thiocyanate levels

Fifty-one of the 60 patients included in the primary metabolic acidosis analysis from the SNP group had analyzable CN records. No whole blood CN levels above the lower limit of quantification were detected in any of the 51 patients. Thiocyanate levels were also low. The maximum recorded urine thiocyanate level was 8.2 mcg/mL, and 37 (66%) of the 56 patients with validated urine thiocyanate measurements had levels that were below the lower limit of quantification. The maximum post-dose plasma thiocyanate level was 3.9 mcg/mL, but the greatest recorded increase from the baseline level was only 1.5 mcg/mL (Table 6).

**Table 6:** Summary of maximum post-dose urine and plasma thiocyanate levels and plasma thiocyanate change score from baseline

	**Urine thiocyanate (mcg/mL)**	**Post-dose plasma thiocyanate (mcg/mL)**	**Plasma thiocyanate change score (mcg/mL)**
N	56	51	34
Mean (SE)	0.85 (0.22)	0.7 (0.2)	0.1 (0.1)
Median (Min, Max)	0.00 (0.00, 8.20)	0.0 (0.0, 3.9)	0.0 (−0.5, 1.5)

N represents the number of participants with urine thiocyanate measures, post-dose plasma thiocyanate measures, or a plasma thiocyanate change score calculated from both pre-dose and post-dose plasma thiocyanate measures.

## Discussion

We performed a case-controlled, retrospective study to evaluate the incidence of metabolic acidosis in children undergoing major surgery and deliberate hypotension with and without SNP. Our findings suggest that, when used for short-term deliberate hypotension, SNP does not cause an increased incidence of metabolic acidosis compared with the use of anesthetic agents alone.

Sodium nitroprusside (SNP) was first discovered in 1850. Its hypotensive effect was reported in 1929, and its first therapeutic use was reported by Page et al. in 1955 [3]. Moraca et al. first described the clinical use of SNP for deliberate hypotension during surgical procedures in 1962 [4]. Since then, SNP has been widely used to control blood pressure in infants and children in the perioperative period.

SNP is a direct-acting vasodilator with a rapid onset of action and rapid offset. Its effect is achieved via release of nitric oxide, thereby activating guanylate cyclase, leading to an increase in intracellular concentrations of cyclic guanosine monophosphate (cGMP). cGMP, in turn, inhibits calcium release from the sarcoplasmic reticulum, resulting in smooth muscle relaxation and vasodilation.

The metabolism of each molecule of SNP results in the liberation of 5 CN molecules (Figure 1). The blood concentration of CN is proportional to the total dose and rapidity of administration of SNP. The CN produced by metabolism of SNP may (1) be converted to thiocyanate in the presence of thiosulfate by the rhodanese system, (2) combine with methemoglobin to produce cyanomethemoglobin or (3) bind with cytochrome oxidase. Binding to cytochrome oxidase is increased when depletion of thiosulfate or rhodanese occurs, resulting in interference with electron transport and oxidative phosphorylation. The latter effect leads to cellular hypoxia, use of anaerobic metabolic pathways and metabolic acidosis due to the production of lactic acid [5]. Increases in CN have been associated with SNP infusion rates > 2 mcg/kg/min. [6,7]. Co-administration of thiosulfate with SNP may prevent CN toxicity; thiosulfate has been co-infused with SNP during deliberate hypotension, although this practice is not routine in most centers^3^[8]. Deaths have been attributed to CN toxicity in children when high doses of SNP have been administered for relatively prolonged periods, but the total dose per unit of time that may be lethal is unknown [9]. It is likely that the use of SNP has been limited because of the assumed association between its use and CN toxicity leading to progressive metabolic acidosis. Accordingly, other hypotensive agents that may be more expensive and less readily titrated than SNP have been used. Until now, no studies with large numbers of patients have been conducted to determine the incidence of metabolic acidosis with intra-operative use of SNP in children.

Yaster et al. compared the efficacy of SNP to nitroglycerine (NTG) in a group of 14 children, ages 9 to 14 years, undergoing craniofacial or spinal fusion surgery [10]. NTG in doses as high as 40 mcg/kg/min was ineffective at decreasing mean arterial pressure (MAP) below 55 mmHg or causing a decrease in MAP greater than one-third of baseline values. SNP was uniformly successful at inducing hypotension in all patients, including those patients in whom NTG failed. The dose of SNP required to induce hypotension was 6–8 mcg/kg/min. The authors concluded that SNP is preferred for “the reliable and sustained induction of deliberate hypotension in children and adolescents.”

Przybylo et al. reported the use of SNP during hypothermic cardiopulmonary bypass in 10 children, ages 1–7 years [11]. SNP was titrated during bypass to maintain a MAP of 35–60 mmHg. The mean dose was 6.0 ± 2.5 mcg/kg/min and the median duration of infusion was 75 min (± 15–90). Blood samples were obtained for whole blood CN and serum thiocyanate concentrations at baseline, every 15 minutes during infusion and up to 24 hours post-infusion. Maximum CN concentrations of ≥ 1 mcg/ml were measured in 2 children, 4 children had maximum values between 0.5 and 1 mcg/ml and CN was not detected in the remaining 4 children. The CN assay technique employed in this study and the accuracy of the results has been questioned [12]. No patient had a significant increase in thiocyanate concentration. Arterial blood gas analysis was performed every 30 minutes during the SNP infusion. No child developed metabolic acidosis, although HCO_3_ 1.8 – 4.6 mEq/kg was added to the cardiopulmonary bypass prime (i.e. prior to SNP administration and blood gas tension recordings) and additional doses of HCO_3_ were given during cardiopulmonary bypass.

Hersey et al. compared SNP and nicardipine infusions during deliberate hypotension for spinal fusion surgery in 20 healthy adolescents [13]. Patients were anesthetized with isoflurane and sufentanil and the study drug was titrated to achieve a MAP of 60 mmHg. Hemodilution to a hematocrit of 25% and intraoperative blood salvage were used. Patients receiving nicardipine had less blood loss than those receiving SNP (761 ± 199 mL vs. 1,297 ± 264 mL, respectively) and had a longer time to restoration of baseline MAP following discontinuation of the study infusion (26.8 ± 4.0 min vs. 7.3 ± 1.1 min, respectively). There were no differences in the amount of IV fluids administered or urine output between the two groups. CN concentrations were not measured and the incidence of metabolic acidosis was not reported. Lustik et al. conducted a similar study comparing SNP and nicardipine for deliberate hypotension in 51 adolescents undergoing spinal surgery and found no difference in blood loss between the groups [14].

There are limitations in each of these studies of SNP. The numbers of patients are small and the incidence of metabolic acidosis is not reported or, in the study by Przybylo et. al., may be masked by the prior administration of HCO_3_. Our study is relatively large but also has several limitations. As in prior studies, the anesthetic agents and doses as well as the volume of IV fluids were not controlled. Targets for MAP were not defined, but rather were determined at the discretion of the anesthesiologist caring for each patient. These parameters are difficult or impossible to control well even in prospective studies due to the overriding concern for patient safety and lack of evidence for the most appropriate endpoints. In part due to the retrospective nature of our study, measurement of arterial blood gas tensions was not performed at pre-defined time points. In the control group, HCO_3_ was administered in some cases prior to the measurement of arterial blood gas tensions.

## Conclusion

We performed a case-controlled, retrospective study of 166 children to evaluate the incidence of metabolic acidosis in children undergoing major surgery and deliberate hypotension with and without SNP. The proportion of patients with low HCO_3_ levels was similar in the control and SNP treatments groups. More patients in the control group needed to have HCO_3_ administered while on anesthesia. No patient to whom SNP was administered had CN levels above the lower limit of quantification, and both plasma and urine thiocyanate concentrations were low. We found no difference in blood loss or IV fluid administration between the two groups, although more blood was administered to patients in the SNP group. Our findings suggest that SNP, when used for short-term deliberate hypotension, does not cause an increased incidence of metabolic acidosis compared with the use of anesthetic agents alone.

## Competing interests

The authors have no competing interests to declare.

## Authors’ contributions

All authors have made substantial contributions to conception and design, and/or acquisition of data, and/or analysis and interpretation of data. All authors have been involved in drafting the manuscript and/or revising it critically for important intellectual content, and have given final approval of the version to be published. All authors read and approved the final manuscript.

## Pre-publication history

The pre-publication history for this paper can be accessed here:

http://www.biomedcentral.com/1471-2253/13/9/prepub
